# Beta-Hydroxybutyric Acid as a Template for the X-ray Powder Diffraction Analysis of Gamma-Hydroxybutyric Acid

**DOI:** 10.3390/molecules29194678

**Published:** 2024-10-02

**Authors:** Domenica Marabello, Carlo Canepa, Alma Cioci, Paola Benzi

**Affiliations:** 1 Department of Chemistry, University of Torino, Via P. Giuria 7, 10125 Torino, Italy; domenica.marabello@unito.it (D.M.); carlo.canepa@unito.it (C.C.); alma.cioci@unito.it (A.C.); 2 Centre for Crystallography (CrisDi), University of Torino, 10125 Torino, Italy

**Keywords:** X-ray powder diffraction, non-destructive analysis, spiked beverages, γ-hydroxybutyric acid (GHB), β-hydroxybutyric acid (BHB), drink analysis

## Abstract

In this paper, we report the possibility of using the X-ray powder diffraction (XRPD) technique to detect gamma-hydroxybutyric acid (GHB) in the form of its sodium salt in different beverages, but because it is not possible to freely buy GHB, beta-hydroxybutyric acid (BHB) and its sodium salt (NaBHB) were used as a model to fine-tune an X-ray diffraction method for the qualitative analysis of the sodium salt of GHB. The method requires only a small quantity of beverage and an easy sample preparation that consists only of the addition of NaOH to the drink and a subsequent drying step. The dry residue obtained can be easily analyzed with XRPD using a single-crystal X-ray diffractometer, which exploits its high sensitivity and allows for very fast pattern collection. Several beverages with different NaBHB:NaOH molar ratios were tested, and the results showed that NaBHB was detected in all drinks analyzed when the NaBHB:NaOH molar ratio was 1:50, using a characteristic peak at very low 2θ values, which also permitted the detection of its presence in complex beverage matrices. Moreover, depending on the amount of NaOH added, shifting and/or splitting of the characteristic NaBHB salt peak was observed, and the origin of this behavior was investigated.

## 1. Introduction

Gamma-hydroxybutyric acid (GHB) is a short-chain fatty acid naturally present in all mammals [1]. It acts as a precursor and metabolite of the inhibitory neurotransmitter gamma-aminobutyric acid (GABA), and it is able to produce sedative, hypnotic, and anesthetic effects [2,3]. The sodium salt of GHB is registered as a therapeutic agent for the treatment of narcolepsy (Xyrem^®^) [4,5] and alcohol dependence to alleviate withdrawal symptoms [6,7]. Nevertheless, over the last few years, due to its chemical–physical, organoleptic, pharmacodynamic, and pharmacokinetic properties, it has begun to be used as a recreational or abuse drug, and its misuse has become a serious social problem, often related to drug-facilitated crimes (DFCs) such as drug-facilitated sexual assault (DFSA) [8,9].

Drug-facilitated crimes are defined as crimes such as robbery, money extortion, cheating, and sexual assault committed while the victim is under the influence of a drug [8,9,10,11,12,13] and, hence, has impaired behavior, perception, or decision-making capacity. Two main circumstances occur in DFC cases: (1) when the perpetrator takes advantage of someone’s voluntary use of alcohol or other drugs (proactive DFC); (2) when the perpetrator intentionally forces someone to consume alcohol or other drugs with or without their knowledge (opportunistic DFC). In this scenario, GHB can be voluntarily ingested for recreational purposes (for example, in a party context or discotheque). In fact, at low doses, it induces a state of euphoria [14,15], disinhibition, and relaxation. Nevertheless, more dramatic effects can occur due to the individual’s sensitivity to the drug [14,16] and because the user is often unaware of the dose they are consuming, considering that the actual quantity of GHB in drugs illegally bought on the street or online is not known. GHB is well suited for criminogenic purposes, such as DFC [9,10,11,12], because it is colorless, odorless, and soluble in both alcoholic and non-alcoholic beverages, easily masking its flavor, and it is rapidly absorbed by the body. Moreover, when the dose is increased, it induces a state of sedation and stupor, allowing the perpetrator to overpower the victim. Finally, it causes a state of retrograde amnesia, preventing the victim from recalling the violence endured [15,17]. For this reason, in many countries, GHB is classified as a controlled substance.

Many papers and reviews [8,9,11,18,19,20,21] have reported on the psychoactive substances used in DFC (and particularly DFSA). Although GHB is not one of the most frequently detected drugs, the possible risk of its involvement must not be overlooked, especially considering that its role in this type of crime may be largely underestimated. In fact, the most common matrices used for forensic purposes are biological (blood, saliva, urine, hair, and others) [22], and GHB can be detected with different analytical techniques [22,23] based on liquid or gas chromatography coupled with mass spectrometry [24,25,26,27,28,29,30], nuclear magnetic resonance spectroscopy [31,32,33,34,35], and IR and Raman spectroscopy [34,35,36,37,38,39,40,41]. Colorimetric, fluorometric, and sensing techniques have also been employed [42,43,44,45]. Nevertheless, GHB has a relatively short half-life and is rapidly metabolized and eliminated from the body, and it is not detectable in blood after 2–8 h or in urine after 8–12 h [46,47,48]. Moreover, there is often a delay of hours or even days between falling victim to and reporting a possible drug-facilitated crime, which can compromise the likelihood of determining exogenous GHB in these kinds of samples.

For these reasons, the true incidence of GHB use in DFC may be higher than previously recognized. Therefore, after filing a police report for proactive or opportunistic GHB-based DFC involving a suspected spiked drink, it is crucial to carefully evaluate the matrices used to collect and analyze GHB in the suspected beverage, if available [49]. For this purpose, different methods have been reported [17,29,30,50,51,52,53,54,55] that, in many cases, require complex sample preparations. For example, with gas chromatography (GC) methods (generally coupled with mass spectrometry, total vaporization solid-phase microextraction, or flame ionization detection), a time-consuming sample preparation procedure is needed, involving extraction, sonication, centrifugation, and derivatization procedures, since GHB cannot be directly analyzed using GC. Moreover, with infrared (IR) spectroscopy, a multi-step sample treatment is required. Methods that employ chemical sensors in colorimetric or fluorescent methods have also been reported in the literature, but they can be affected by poor selectivity.

A complicating factor is the interconversion of GHB to its lactone form (gamma-butyrolactone, GBL), which is pH-dependent, meaning that the more acidic the conditions, the more GHB converts to GBL, while under strongly alkaline conditions, GBL is completely converted back to GHB [36,56].

Among the analytical techniques for forensic purposes, X-ray powder diffraction (XRPD) is an attractive method for the characterization of crystalline and semi-crystalline materials [57,58,59,60,61], and it shows great promise for analyzing both new and traditional street samples of psychoactive substances [62,63,64]. XRPD allows for non-destructive analysis and can distinguish between substances with similar chemical structures, both of which are fundamental requirements in the forensic analysis of psychoactive substances.

The aim of this work was to propose a new XRPD method for the qualitative analysis of GHB in beverages using a diffractometer built for single-crystal diffraction.

The most used XRPD diffractometers, equipped with point detectors, require grinding of samples to assure the casual orientation of crystals and to obtain good reproducibility of the measurements. By contrast, faster CCD area detectors, normally mounted on diffractometers for single-crystal diffraction, allow the calculation of peak intensities by integrating over all diffracted circles, enabling the collection of reproducible patterns even for oriented or unground samples. Moreover, these instruments are characterized by highly sensitive CCD detectors and intense, highly focused X-ray beams, allowing for the collection of high-quality powder diffraction patterns even from very small quantities of material. This capability is particularly useful for the non-destructive analysis of GHB in dried residues from adulterated beverages used in DFC.

GHB and its lactone form are present in the list of substances subjected to D.P.R. n. 309/90 of the Italian Ministry of Health [65,66], and their use and sale are strictly regulated by law. Thus, it is not possible to freely buy them. Consequently, in this study, β-hydroxybutyric acid (BHB) and its sodium salt (NaBHB) were used as models to fine-tune a method for qualitative analysis of different beverages using X-ray diffraction. BHB and NaBHB are chemical analogs of GHB and NaGHB, respectively, and are expected to exhibit similar chemical behaviors.

The method developed uses a very easy sample preparation and allows rapid qualitative analysis to be performed. Moreover, if used for GHB (NaGHB) analysis, it prevents the problems connected with GHB–GBL interconversion [36,56].

## 2. Results and Discussion

As reported in the Section 3, to test the reproducibility of the method, each sample was tested three times, and each time, the same results were obtained.

**Figure 1:**
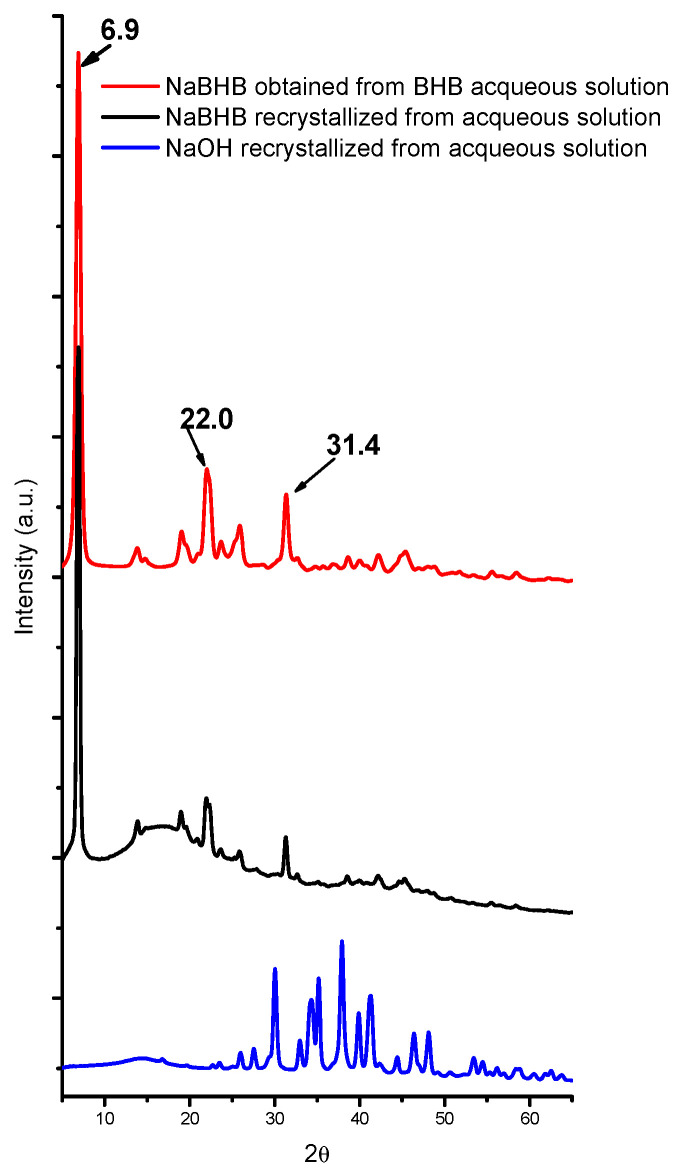
The XRD pattern of NaBHB obtained from BHB + NaOH in an aqueous solution and NaBHB and NaOH recrystallized from aqueous solutions. The patterns have been translated on the ordinate axis and normalized with respect to the higher signal of NaBHB.

Figure 1 shows the two powder patterns of the NaBHB standard, obtained using the two procedures described in the Section 3.2. The pattern of NaOH recrystallized from an aqueous solution is also shown. Comparing the standard patterns to that of the recrystallized NaOH reveals that only the peaks of NaBHB are present, even for the standard obtained from a BHB + NaOH solution.

Considering that the very large band visible in the background of the patterns in the 2θ range of 8.5 ÷ 20° is attributable to added paraffin, the XRPD patterns for the two standards are identical. The NaBHB pattern is characterized by a high, sharp peak at 2θ = 6.9°, with less intense and more superimposed peaks at higher 2θ values.

For NaGHB, a similar pattern was observed [67], with a high, sharp peak at 2θ = 6.16°, and this fact supports the use of BHB and NaBHB as models to fine-tune a method for the XRPD analysis of GHB and NaGHB.

Series A samples. To verify the possibility of detecting NaBHB using XRPD for qualitative analysis of beverage residues, a preliminary analysis on three samples—coke, scotch, and red wine (non-alcoholic and with high and low alcohol contents, respectively) spiked with a considerable amount of BHB (20% *v*/*v*) and a 1:1 BHB:NaOH molar ratio—was performed using the procedure described in the Section 3.

**Figure 2:**
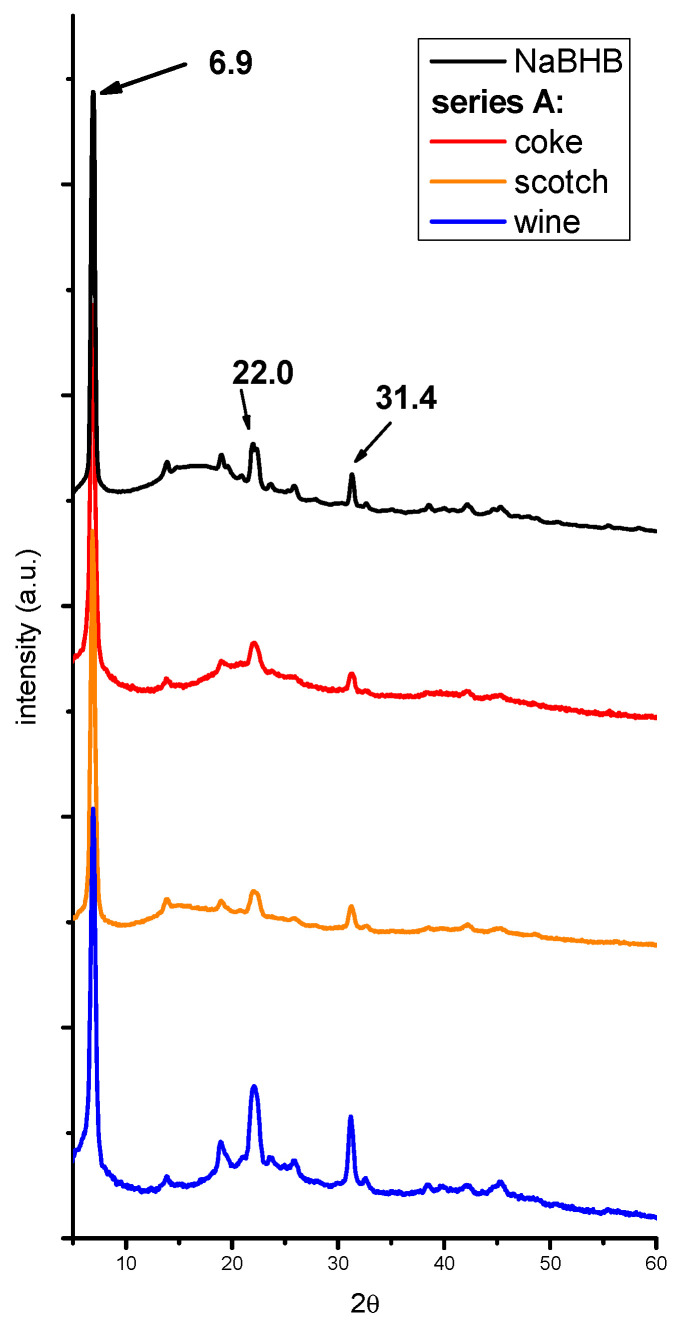
The XRPD patterns of the solids obtained from different beverages spiked with 20% *v*/*v* of BHB and a 1:1 BHB:NaOH ratio (series A). The pattern of NaBHB obtained from the BHB aqueous solution is also reported for comparison. The patterns have been translated on the ordinate axis and normalized with respect to the higher signal of NaBHB.

**Figure A1:**
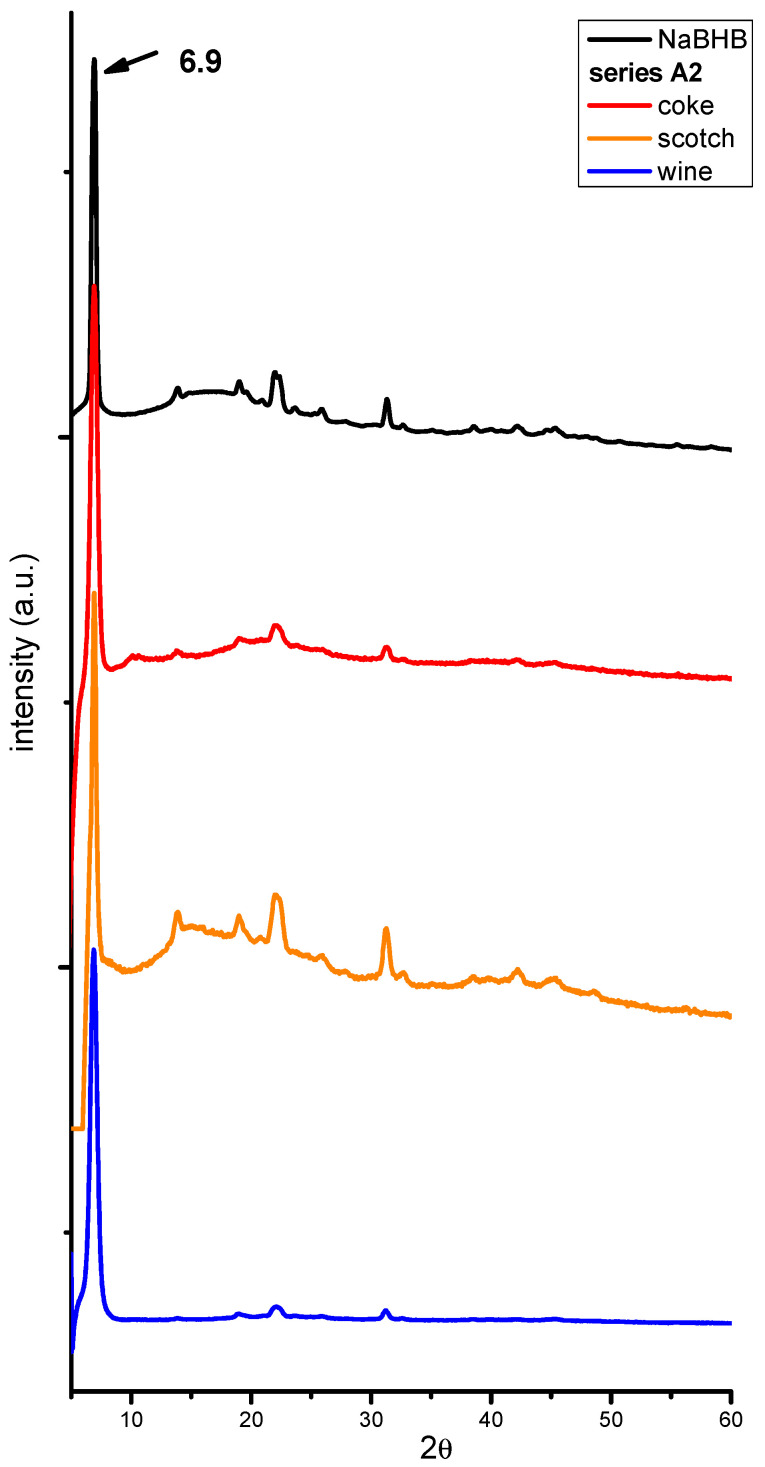
The XRPD patterns of the solids obtained from different beverages spiked with 20% *v*/*v* NaBHB and a 1:1 NaBHB:NaOH molar ratio. The pattern of NaBHB recrystallized from an aqueous solution is also reported for comparison.

The XRPD patterns of the dry residues are reported in Figure 2. For all samples, a low-angle peak of NaBHB is clearly visible, indicating that BHB (in the NaBHB form) can be easily detected. The same results were obtained when the beverages were spiked with NaBHB (series A2), as shown in Figure A1 of Appendix A.

**Figure 3:**
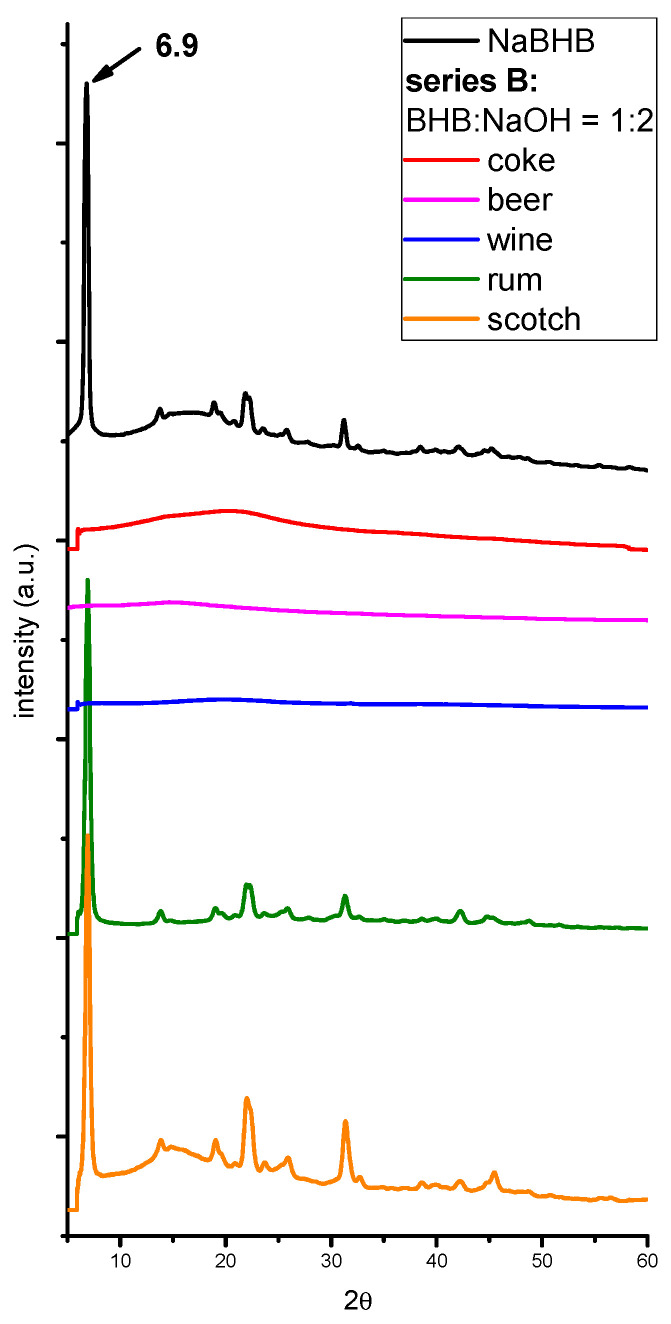
The XRPD patterns of the residues of some beverages spiked with 1% *v*/*v* of BHB and a 1:2 BHB:NaOH ratio (series B1). The pattern of the standard NaBHB obtained from the BHB aqueous solution is also reported for comparison. The patterns have been translated on the ordinate axis and normalized with respect to the higher signal of NaBHB.

**Figure A2:**
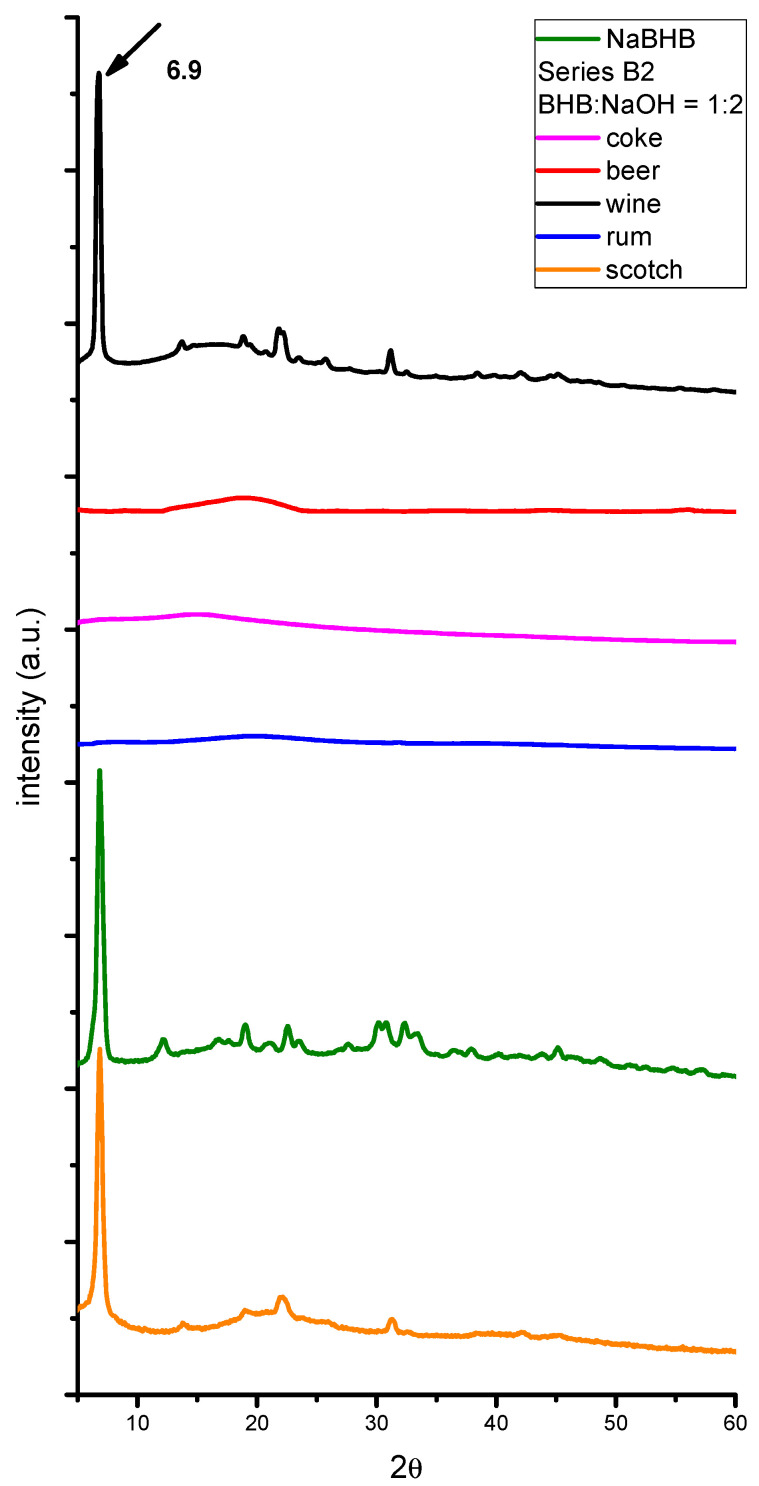
The XRPD patterns of the residues of some beverages spiked with 1% *v*/*v* NaBHB and a 1:2 NaBHB:NaOH molar ratio. The pattern of NaBHB recrystallized from an aqueous solution is also reported for comparison. The patterns have been translated on the ordinate axis and normalized with respect to the higher signal of NaBHB.

Series B1 samples. For illicit purposes, the quantities usually added to beverages range from 1 to 5 g of NaGHB or from 2 to 10 mL of pure GHB to a volume of 100 or 200 mL of drink [14,15,16,17,29,50]. Therefore, a second series of samples (series B1) of several common beverages (coke, beer, rum, scotch, and wine) was analyzed. These samples were spiked with a lower BHB percentage (1% *v*/*v*) and a 1:2 stoichiometric BHB:NaOH molar ratio to favor the formation of sodium salt. The X-ray analyses of the dry residues, shown in Figure 3, reveal signals attributable to NaBHB only in scotch and rum. In agreement with the results obtained from the series B3 samples (see below), no peaks from other crystalline compounds are present. Similar results (shown in Figure A2 of Appendix A) were obtained with beverages directly spiked with 1% *w*/*v* of NaBHB (series B2 samples).

**Figure A3:**
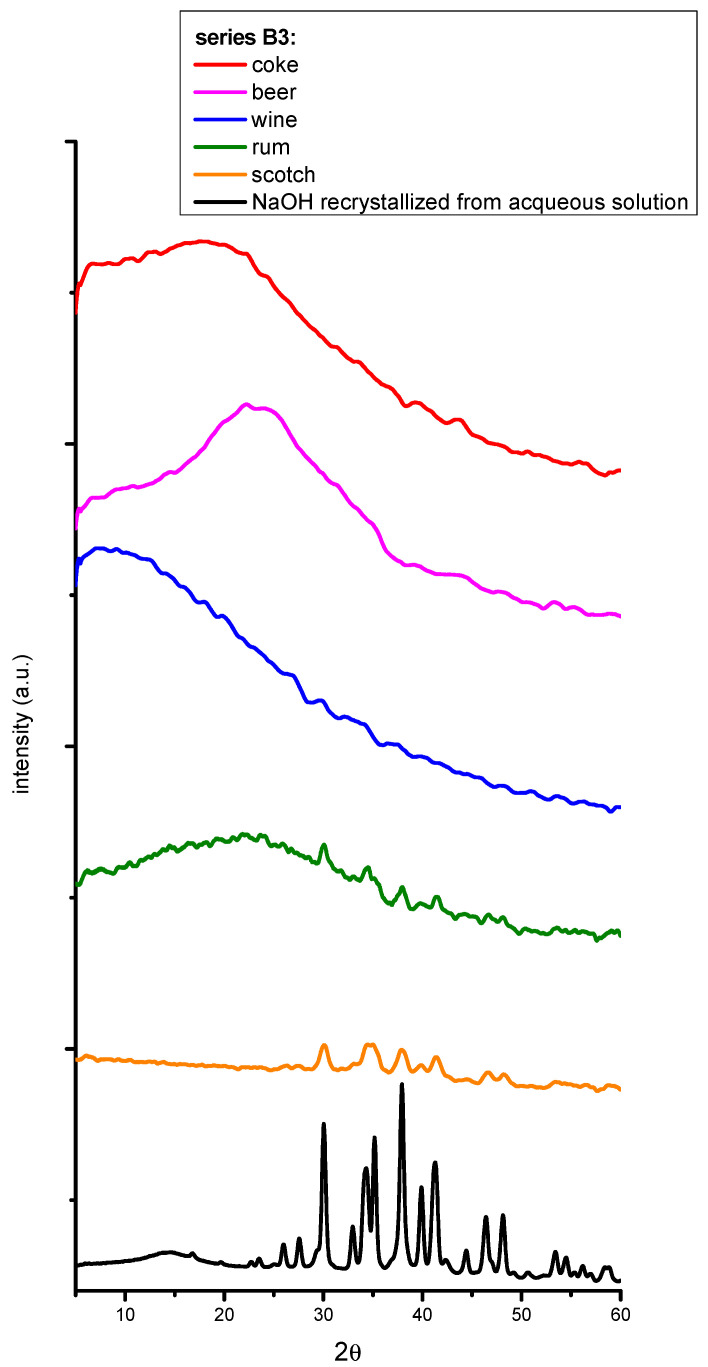
The XRPD patterns of the residues of some beverages without BHB but spiked with the same NaOH content of series B samples. The pattern of NaOH recrystallized from aqueous solution is also re-ported for comparison. The patterns have been translated on the ordinate axis.

Series B3 samples. Additionally, samples of the same beverages but without BHB and with the same NaOH content as the series B samples were prepared to determine whether naturally occurring GHB [30,68,69] or other chemical substances in the beverage matrix could form crystalline compounds after drying. The XRPD patterns, shown in Figure A3 of Appendix A, do not evidence any peaks from crystalline species, except for the rum and scotch samples, which show only weak signals attributable to NaOH.

To explain the absence of a NaBHB signal for the series B1 coke, beer, and wine samples, two hypotheses can be considered. The first hypothesis is a low sensitivity of the analytical method. It should be noted that (i) after the drying step, only a small amount of the residue is needed for X-ray diffraction analysis; (ii) due to the varying compositions of the beverages, different amounts of dry residue (g/mL) are obtained, resulting in significant variation in the NaBHB percentage relative to the total weight of the residues across different beverages.

Table 1 reports the amount of dry residue obtained for the non-spiked beverages and the calculated percentage of NaBHB relative to the total weight of the residues for the spiked samples with 20% *v*/*v* of BHB and a 1:1 BHB:NaOH molar ratio. The percentages for the spiked beverages with 1% *v*/*v* of BHB and a 1:2 or 1:50 BHB:NaOH molar ratio are also reported.

As expected, the different non-spiked beverages demonstrated different dry weights; therefore, when BHB was added, the percentage of NaBHB in the dry residue varied widely.

The series A samples were spiked with a high amount of BHB, resulting in a very high percentage of NaBHB in the dry residue (from 72.3% to 96.3%), allowing for easy detection. Moreover, the data in Table 1 show that even for the rum and scotch in series B, the percentage of NaBHB is high (higher than 65%). In contrast, for coke, beer, and red wine, it is less than 31%. As reported above, NaBHB was detected in the rum and scotch samples but not in the coke, beer, and red wine. Therefore, it can be hypothesized that the low percentage of NaBHB in the dried residues of some of the spiked beverages with 1% BHB might explain the negative results.

The second hypothesis is that these negative results may be due to secondary reactions between NaOH and other substances in the beverage matrices, which prevent the reaction between BHB and NaOH needed to form NaBHB. In fact, beverages contain various acidic substances, such as carbonic acid in fizzy drinks, phosphoric acid in coke, and carboxylic acids in alcoholic drinks [70,71], which can neutralize the added NaOH, preventing its reaction with BHB. Moreover, because BHB is a weak acid, its equilibrium with its deprotonated form is shifted toward the BHB form in the presence of acidic substances.

In the beverages spiked with 1% BHB and a 1:2 BHB:NaOH molar ratio, the amount of NaOH was small and likely consumed by secondary reactions, leaving insufficient NaOH for the salification of BHB. This hypothesis is supported by the XRPD patterns of the series B samples, where NaOH peaks were not visible despite NaOH being in excess relative to the amount of BHB. In contrast, the beverages spiked with 20% BHB had a higher NaOH concentration, with a 1:1 BHB:NaOH molar ratio. It is reasonable to hypothesize that, even after accounting for matrix reactions, some NaOH remains available for the salification reaction.

**Figure 4:**
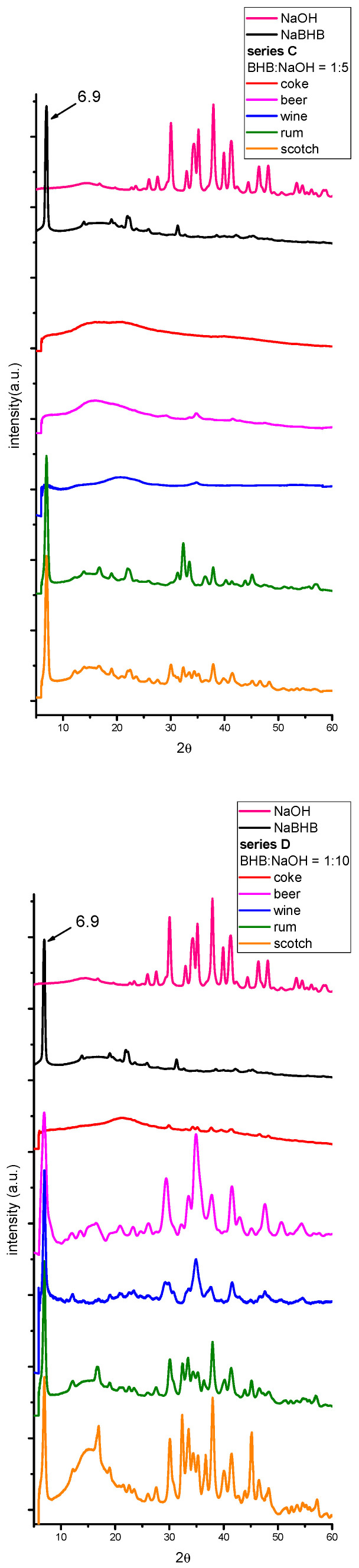

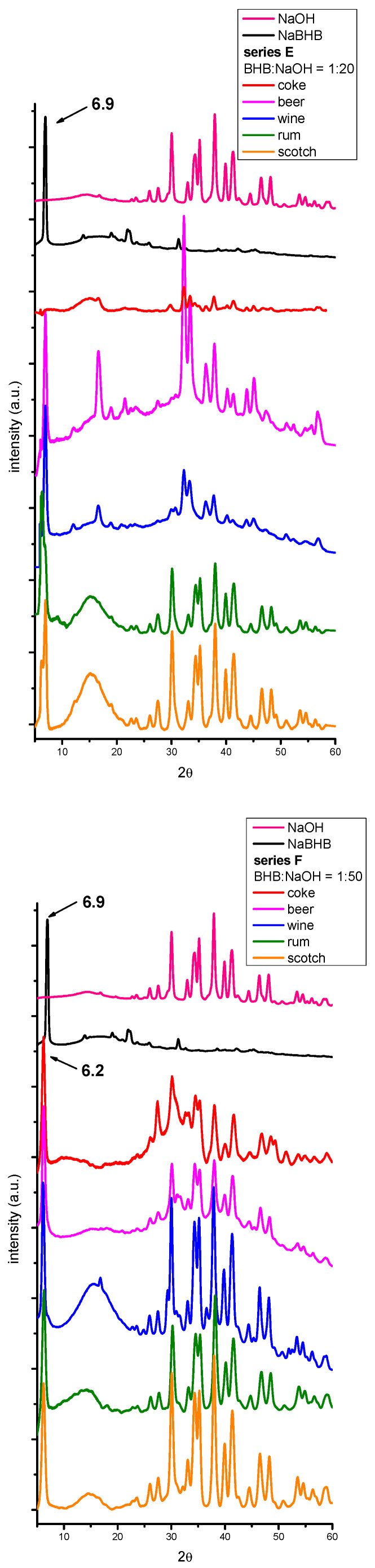
The X-ray patterns of the solids obtained from some beverages spiked with 1% *v*/*v* BHB and a BHB:NaOH ratio of 1:5 (series C), 1:10 (series D), 1:20 (series E), and 1:50 (series F). The patterns of NaBHB obtained from the BHB aqueous solution and of recrystallized NaOH are also shown. The patterns have been translated on the ordinate axis and normalized with respect to the higher signal of NaBHB.

Series C, D, E, and F samples. To explore the second hypothesis, different series of samples with 1% BHB and BHB:NaOH molar ratios, 1:5, 1:10, 1:20, and 1:50 (series C, D, E, and F, respectively), were prepared. The XRPD patterns are shown in Figure 4. As for the series B samples, only the rum and scotch samples from series C (a BHB:NaOH molar ratio of 1:5) showed NaBHB signals. As the NaOH content further increased (from series C to F), the XRPD patterns became more complicated due to the appearance of NaOH signals. When the BHB:NaOH molar ratio reached 1:50, peaks over 2θ = 20° were attributable to NaOH. Consistent with the results from the series B3 samples, no peaks from other crystalline compounds were observed.

Nevertheless, the most intense peak at low 2θ values, characteristic of NaBHB, is clearly visible when the salt is present. Again, the large band in the 2θ range between 8.5° and 20°, visible in the patterns of some samples in Figure 4, is attributable to the paraffin used to compact the powders.

Moreover, as described in the Section 3, the samples were not ground before the analysis, and hence, the distribution of NaBHB and NaOH in the solid residues was not uniform. For this reason, the ratios between the higher peak of NaBHB and that of NaOH did not follow a regular trend when the NaOH amount was increased.

Furthermore, the results indicate that beverages with more complex matrices and a higher amount of dry residue (see Table 1) require a greater amount of NaOH to form NaBHB. Figure 4 evidences that as the NaOH content was increased, the low-angle peak of NaBHB became more pronounced, even in the XRPD patterns of the beer and wine samples of series D and E and in all samples of series F. This finding contradicts the first of the previously reported hypotheses: as the NaOH content increases, the NaBHB weight percentage relative to the total weight of the dry residue decreases. For example, in the scotch and coke samples, when the BHB:NaOH molar ratio varied from 1:2 to 1:50, the NaBHB weight percentage dropped from 71.6% to 5.8% and from 11.2% to 4.1%, respectively (see Table 1). Additionally, it is noteworthy that, when NaBHB was not detected, the XRPD pattern also showed an absence of NaOH signals (in the coke, beer, and wine from series C) or very weak signals (in the coke of series D and E).

Thus, the results obtained are in agreement with the second hypothesis; that is, the negative results (the absence of NaBHB signals in the XRPD pattern of the dried residues of some beverages spiked with a low amount of NaOH) could be due to secondary reactions of NaOH with other substances in the beverage matrices, thus preventing the reaction between BHB and NaOH.

**Figure 5:**
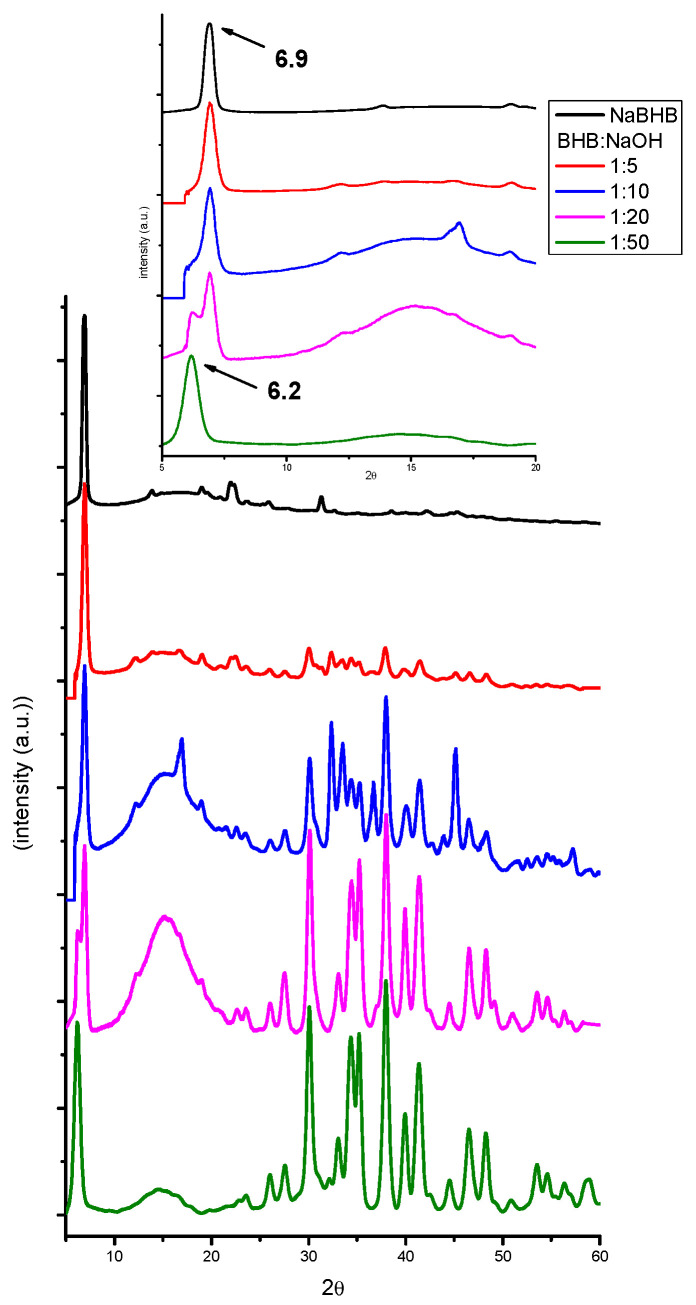
The X-ray patterns of dry residues from the scotch samples spiked with 1% *v*/*v* BHB and BHB:NaOH ratios 1:5, 1:10, 1:20, and 1:50. The inset highlights the region of 2θ values between 5° and 20°. The patterns have been translated on the ordinate axis and normalized with respect to the higher signal of NaBHB.

**Figure A4:**
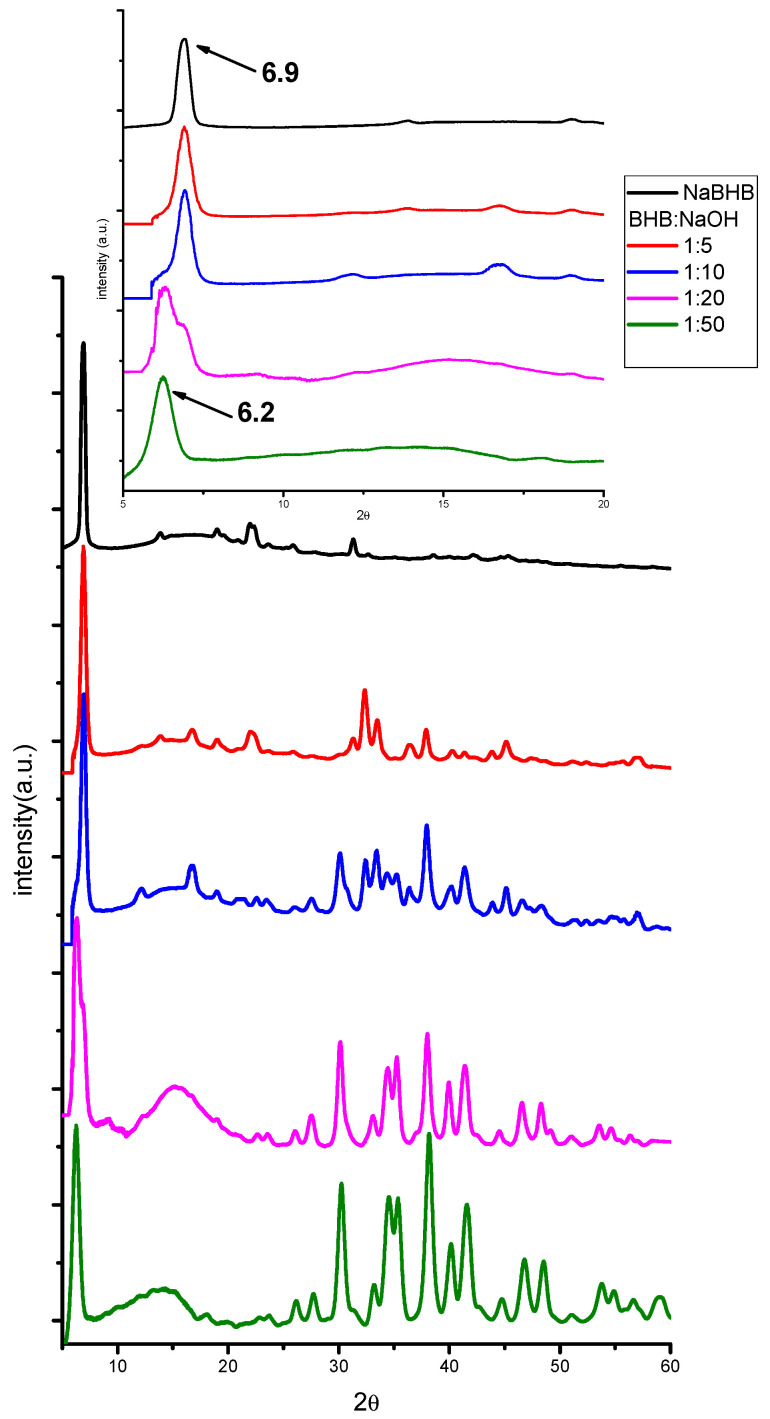
The X-ray patterns of dry residues from the rum samples spiked with 1% *v*/*v* BHB and BHB:NaOH molar ratios of 1:5, 1:10, 1:20, and 1:50. The inset highlights the region between 5° and 20° 2θ values. The patterns have been translated on the ordinate axis and normalized with respect to the higher signal of NaBHB.

Another interesting observation is the shift in the position of the highest peak of NaBHB with the increasing NaOH amount. For BHB:NaOH molar ratios up to 1:10, the peak is positioned at 6.9°, while at a BHB:NaOH molar ratio of 1:50, it shifts to ca. 6.2°. Additionally, for a BHB:NaOH molar ratio of 1:20, the peak for the rum and scotch samples splits into two signals at 6.2° and 6.9°. Figure 5 shows the XRPD patterns of the scotch samples at different BHB:NaOH molar ratios to illustrate this behavior. The inset highlights the region between 5° and 20° 2θ values. The XRPD patterns of the rum samples are shown in Figure A4 of Appendix A.

**Figure 6:**
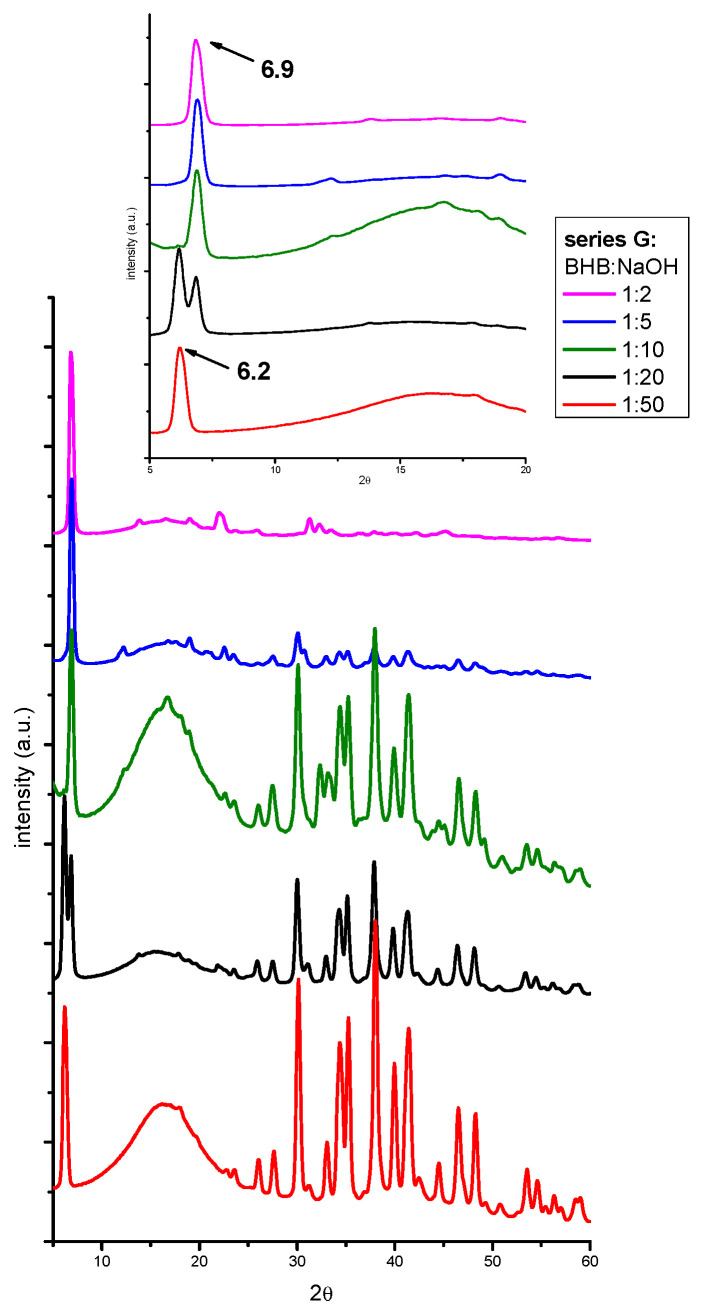
The X-ray patterns of the dry residues obtained from water solutions spiked with 1% *v*/*v* BHB and BHB:NaOH ratios from 1:2 to 1:50. The inset highlights the region of 2θ values between 5° and 20°. The patterns have been translated on the ordinate axis and normalized with respect to the higher signal of NaBHB.

Series G samples. To understand these results, a series of water solutions with 1% BHB and different BHB:NaOH molar ratios (from 1:2 to 1:50) (series G) was prepared. The results of the XRPD analyses are shown in Figure 6. The inset highlights the region of 2θ values between 5° and 20°.

Even in this case, in solutions with BHB:NaOH molar ratios of 1:2, 1:5, and 1:10, the highest NaBHB peak was found at 2θ = 6.9°. Furthermore, in the solution with a 1:20 molar ratio, a signal at 6.2° appeared, while at a 1:50 molar ratio, the 6.9° peak disappeared, leaving only the signal at 6.2°. This behavior suggests the possible formation of either a hydrated form of NaBHB or two polymorphs with similar structures, depending on the NaOH concentration, with the transition from one to the other occurring as the NaOH content increases.

Series H samples. Considering the above results, the XRPD technique was used to analyze different beverage samples (series H) spiked with 1% NaBHB (*w*/*v*) and a NaBHB:NaOH molar ratio of 1:50. 

**Figure 7:**
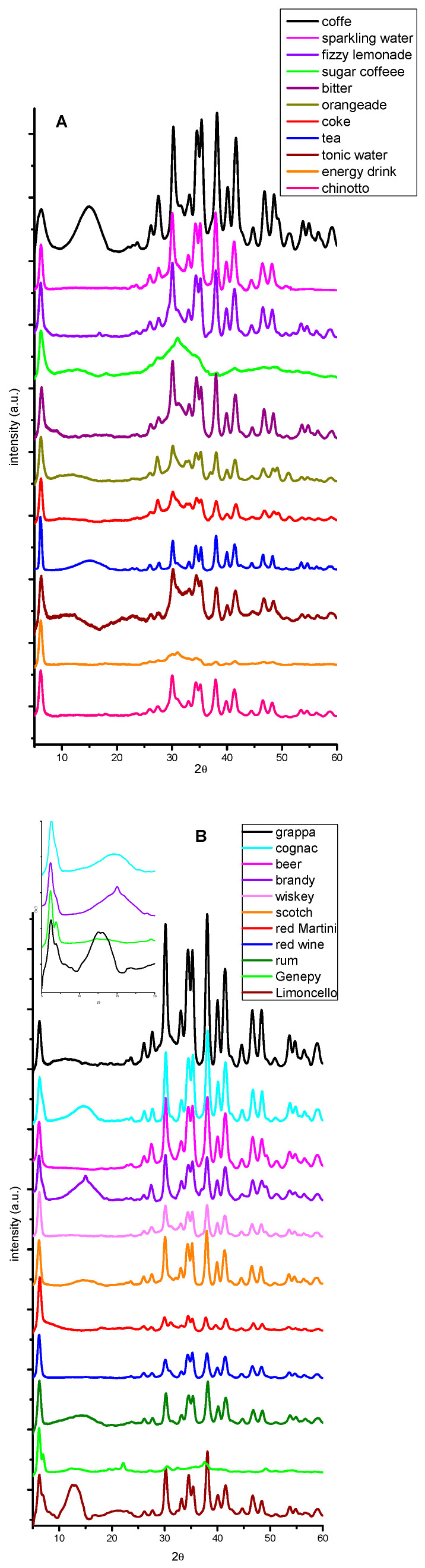

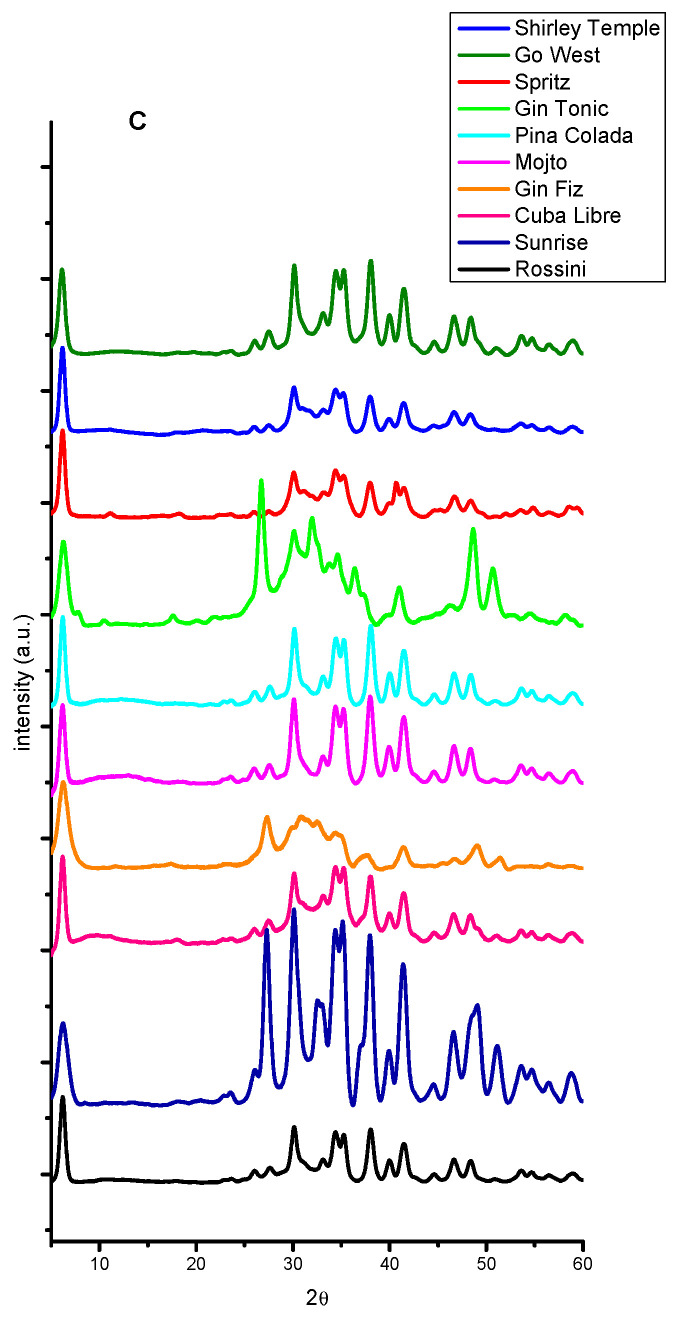
The XRPD patterns of some non-alcoholic (**A**), alcoholic (**B**), and cocktail beverages (**C**). The patterns have been translated on the ordinate axis and normalized with respect to the higher signal of NaBHB.

The tested beverages are listed in Table 2 and the results of XRPD analysis are shown in Figure 7A–C.

As can be observed in the powder patterns in Figure 7, a characteristic signal at 2θ = 6.2° is clearly visible in all the beverages tested, indicating that NaBHB can be qualitatively detected in all the analyzed drinks under these experimental conditions. Nevertheless, it must be noted that, for some samples (cognac, brandy, Genepy, and Limoncello), a signal at 2θ = 6.9° is also present (as evidenced in the inset of Figure 7B), indicating that a hydrated form of NaBHB or both suspected polymorphs are present simultaneously.

## 3. Materials and Methods

### 3.1. Materials

Sodium hydroxide, β-hydroxybutyric acid (BHB) as a 95% (*w*/*w*) solution with a density of 1.126 g/mL, and sodium β-hydroxybutyrate (NaBHB) were purchased from Sigma-Aldrich.

The alcoholic and non-alcoholic beverages were purchased from local supermarkets and cocktail bars.

### 3.2. Standard Preparation

In order to perform diffraction analysis, the sample must be in a solid and crystalline form; thus, X-ray diffraction is generally not a suitable technique to analyze drugs dissolved in beverages. BHB, as an analog of GHB, is a molecular liquid, but it can be easily converted to a solid state by adding a base, such as NaOH, to form NaBHB salt. Thus, to compare the XRPD patterns of the samples, the XRPD pattern of the standard NaBHB salt, which is not available in the literature, was first required. NaBHB salt was synthesized from a BHB aqueous solution following the same procedure adopted by Gorecho et al. [72] for obtaining NaGHB from GHB. An equimolar quantity of BHB was added to a 5.0 M solution of NaOH, and the solution was dried at 50 °C until a precipitate formed. Finally, the precipitate was filtered, washed with acetone, and rapidly dried before collecting the diffraction pattern. Since GHB is sometimes introduced directly by the perpetrator into the adulterated beverages in its salt form, the diffraction pattern of the purchased NaBHB, recrystallized from its aqueous solution, was also collected.

### 3.3. Sample Preparation

To perform XRPD analysis on liquid beverages, the samples need to be converted into solid form. This work developed a simple preparation method that involves two steps: first, adding a base (NaOH) to neutralize the BHB molecule in the drink, and second, drying the beverage to obtain the drug in a solid crystalline form (NaBHB salt) within the dried residue. Considering that during a law enforcement investigation, the amount of beverage collected may be very small—consisting only of the residue remaining after drinking—each beverage sample was limited to just 50 μL.

The tested beverages are listed in Table 2. The cocktail recipes are reported in Appendix A.

The spiked beverage solutions were prepared by adding the appropriate quantity of the drug to 50 μL of each beverage and then adding NaOH to induce the formation of BHB sodium salt. After manual stirring, the solutions were dried, and a small portion of the residue was directly analyzed using XRPD without other manipulations. Each beverage was tested three times. The samples were not ground before XRPD analysis. To avoid moisture absorption, after drying, the samples were stored in an oven at 60 °C or in capped vials.

Sample Series. Solutions containing 20% and 1% (*v*/*v*) BHB or 1% (*w*/*v*) NaBHB were used, and different BHB:NaOH (or NaBHB:NaOH) molar ratios were tested (from 1:1 to 1:50).

Series A samples. The first series of samples consisted of coke, scotch, and red wine containing 20% *v*/*v* BHB. To promote the transformation of BHB into sodium salt, sodium hydroxide was added to achieve a 1:1 stoichiometric BHB:NaOH molar ratio before the drying step.

Series A2 samples. Coke, scotch, and red wine spiked with 20% *w*/*v* NaBHB and NaOH to achieve a 1:1 stoichiometric NaBHB:NaOH molar ratio.

Series B1 samples. Coke, beer, rum, scotch, and red wine containing 1% *v*/*v* BHB and NaOH to achieve a 1:2 stoichiometric BHB:NaOH molar ratio.

Series B2 samples. Coke, beer, rum, scotch, and red wine containing 1% *w*/*v* NaBHB and NaOH to achieve a 1:2 stoichiometric NaBHB:NaOH molar ratio.

Series B3 samples. Coke, beer, rum, scotch, and red wine without BHB but with the same NaOH content as the series B samples.

Series C, D, E, and F samples. Coke, beer, rum, scotch, and red wine containing 1% *v*/*v* BHB and NaOH to achieve 1:5, 1:10, 1:20, and 1:50 stoichiometric BHB:NaOH molar ratios, respectively.

Series G samples. Water solutions with 1% BHB *v*/*v* and NaOH to achieve 1:2, 1:5, 1:10, 1:20, and 1:50 stoichiometric BHB:NaOH molar ratios, respectively.

Series H samples. Beverage samples listed in Table 2 spiked with 1% NaBHB (*w*/*v*) and NaOH to achieve a NaBHB:NaOH molar ratio of 1:50.

### 3.4. X-ray Powder Diffraction (XRPD)

Due to the small mass of the samples analyzed, a highly sensitive XRPD instrument is needed. Therefore, an instrument normally used for single-crystal X-ray diffraction was chosen. This instrument features a very sensitive area detector, which enables the collection of reproducible XRPD patterns even in the presence of the preferred orientation and/or granularity of the sample. Since the solid residue from beverages is often hygroscopic and too small for grinding, the area detector is essential for obtaining reproducible XRPD patterns.

The X-ray powder diffraction (XRPD) patterns were collected at room temperature using an Atlas S2 Rigaku-Oxford Diffraction Gemini R-Ultra diffractometer equipped with mirror monochromatized Cu-Kα (1.5418 Å) radiation. A small portion of the dried powdered sample was compacted and modeled as a ball ca. 0.45 mm in diameter (less than the diameter of the X-ray beam). In some cases, with powders challenging to compact, the sample was compacted with a little drop of paraffin oil (non-drying immersion oil for microscopy, type B, code 1248, Cargille Laboratories). The ball was placed on the tip of a glass capillary, which was mounted on the goniometer head of the instrument. Each powder pattern was collected by rotating the sample through 60°, with an exposure time of 60 s.

## 4. Conclusions

In this study, an XRPD technique to detect the presence of BHB and/or NaBHB in a range of non-alcoholic and alcoholic beverages was developed. NaBHB was detected in all drinks analyzed with a NaBHB:NaOH molar ratio of 1:50. For different NaBHB:NaOH ratios, the typical low-angle NaBHB peak was not detected in all samples, probably due to NaOH’s reaction with the sample’s matrix, preventing the formation of NaBHB salt. This hypothesis is supported by the fact that beverages with a higher weight of dry residues needed a higher amount of NaOH to obtain a positive result.

It must also be noted that the characteristic low-angle peak of NaBHB salt was found at 2θ = 6.9° for samples with a 1:2 NaBHB:NaOH molar ratio, shifting to ~6.2° for samples with a 1:50 NaBHB:NaOH molar ratio, and splitting in both positions for intermediate NaBHB:NaOH molar ratios. This behavior suggests that, at higher NaOH concentrations, a hydrated or different polymorph of NaBHB is formed with a similar crystalline structure.

The method developed in this work has the great advantage of needing only a very small amount of beverage (50 μL) and involves a simple preparation method: just the addition of NaOH and a drying step. Furthermore, employing a highly sensitive instrument such as a single-crystal diffractometer eliminates the need for sample grinding and allows for the rapid collection of XRPD patterns. This enables quick analysis results, which is of fundamental importance during forensic investigations.

Since BHB is chemically similar to GHB, a drug used for drug-facilitated crimes, it is presumed that the results obtained in this work can be transferred to the analysis of GHB in the same beverages. In this case, an additional advantage of this method over previous analysis techniques is that it avoids GHB–GBL interconversion [36,56], as a strongly basic pH is used to prepare the dry crystalline residue.

In conclusion, in this study, an X-ray diffraction method for the qualitative analysis of NaBHB was proposed, using a single-crystal diffractometer to analyze a small amount of a sample. Although single-crystal X-ray diffraction instruments are well suited for forensic analysis, they are not widely used in analytical laboratories, likely due to their high cost. However, many universities and research institutes are equipped with these instruments, and scientific police often use these facilities. For our purposes, we selected the single-crystal instrument over traditional instruments for XRPD analysis because it allows for working with small sample amounts, which requires a very sensitive XRPD instrument. Nevertheless, if larger quantities of beverage samples are available, traditional X-ray diffractometers can also be employed with the method proposed in this work.

Future research will involve the development of an X-ray diffraction method for the quantitative analysis of NaBHB in different beverages. Building on the results of this study, a potential approach for quantitative analysis could involve adding an internal standard to the spiked beverage before the drying step. This will be explored in our next study.

## Figures and Tables

**Table 1:** Tested beverages, the dry residue (g/mL) of non-spiked beverages, and the NaBHB weight percentage relative to the total weight of the residues obtained after drying the spiked samples with 1% and 20% *v*/*v* BHB and different BHB:NaOH molar ratios.

Beverage	Dry Residue (g/mL) (Non-Spiked)	NaBHB Weight Percentage (*w*/*w*)		
		BHB 1% *v*/*v* BHB:NaOH 1:2	BHB 20% *v*/*v* BHB:NaOH 1:1	BHB 1% *v*/*v* BHB:NaOH 1:50
Coke	0.099	11.2	72.3	4.1
Beer	0.037	23.9		5.2
Red wine	0.025	30.7	91.2	5.4
Rum	0.002	67.8		5.5
Scotch	0.001	71.6	96.3	5.8

**Table 2:** Tested beverages. ABV, alcohol by volume (%) content.

Non-Alcoholic	Alcoholic	Cocktail
Coffee (Nespresso)	White wine schnapps (Candolini, 40.0 ABV)	Shirley Temple
Sparkling water (Sparea—Pontevecchio)	Cognac (Couvoisier, 40.0 ABV)	Go West
Fizzy lemonade (Lemonsoda—Royal Unibrew)	Beer (Carlsberg, 5.0 ABV)	Aperol spritz
Sugary coffee (Nespresso)	Brandy (Vecchia Romagna, 37.2 ABV)	Gin and tonic
Bitter (Crodino—Campari Group)	Whiskey (Jack Daniel’s, 40.0 ABV)	Piña colada
Orangeade (Fanta—Coca-Cola HBC Italia)	Scotch (J&B, 40.0 ABV)	Mojito
Coke (Coca Cola—Coca-Cola HBC Italia)	Red Martini (14.4 ABV)	Gin fizz
Tea (Lipton)	Red wine (Dolcetto, 13.5 ABV)	Cuba libre
Tonic water (Schweppes—San Benedetto)	Rum (Havana Club, 40.0 ABV)	Sunrise
Energy drink (Red Bull—Rauch)	Genepy (Alpe Herbetet, 38.0 ABV)	Rossini cocktail
Chinotto (Lurisia)	Limoncello (Limoncè, 25.0 ABV)	

## Data Availability

The original contributions presented in the study are included in the article; further inquiries can be directed to the corresponding authors.

## Appendix A

Cocktail recipes.
Shirley Temple:
20 mL grenadine;
100 mL ginger ale;
1 lemon slice;
Ice cubes.
Go West:
30 mL Limoncello;
30 mL white wine;
15 mL hazelnut Irish cream;
15 mL simple syrup;
15 mL lemon juice.
Aperol spritz:
60 mL prosecco;
1 splash club soda;
44 mL Aperol;
1 slice orange;
Ice cubes.
Gin and tonic:
50 mL gin;
150 mL tonic water;
1 lemon;
Ice cubes.
Piña colada:
50 mL white rum;
30 mL coconut cream;
50 mL fresh pineapple juice;
Ice cubes.
Mojito:
10 fresh mint leaves;
44 mL white rum;
½ medium lime;
2 tablespoons white sugar;
120 mL club soda;
Ice cubes.
Gin Fizz:
45 mL gin;
30 mL fresh lemon juice;
10 mL simple syrup;
Splash of soda water;
Ice cubes.
Cuba libre:
50 mL white rum;
120 mL Coca Cola;
10 mL fresh lime juice;
Ice cubes.
Sunrise:
90 mL orange juice;
60 mL sparkling water;
7 mL grenadine;
Ice cubes.
Rossini:
50 g strawberries;
10 g sugar;
100 mL prosecco.
